# Behavioral and Physiological Evidence for Palp Detection of the Male-Specific Attractant Cuelure in the Queensland Fruit Fly (*Bactrocera tryoni*)

**DOI:** 10.3389/fphys.2018.00990

**Published:** 2018-07-26

**Authors:** Thomas A. Verschut, Kevin Farnier, J. Paul Cunningham, Mikael A. Carlsson

**Affiliations:** ^1^Department of Zoology, Stockholm University, Stockholm, Sweden; ^2^AgriBio, Department of Economic Development, Jobs, Transport and Resources, Bundoora, VIC, Australia

**Keywords:** *Bactrocera tryoni*, cuelure, olfactory, maxillary palp, pest management, attractant

## Abstract

The Queensland fruit fly, *Bactrocera tryoni*, is considered one of the worst horticultural pests in Australia attacking a large variety of fruit crops. To defeat pest insects, olfactory attractants have been developed and widely used in lure and kill strategies. Male *B. tryoni* are strongly attracted to the compound raspberry ketone and its synthetic analog, cuelure. Despite the strong behavioral response, a recent study failed to show any activation of antennal receptors to cuelure. Therefore, we hypothesized that cuelure may be detected by an accessory olfactory organ, the maxillary palp. Combining behavioral and physiological experiments we clearly demonstrate that male flies, but not female flies, primarily use the maxillary palps and not the antennae to detect and respond to cuelure. Furthermore, regardless of satiety status, male flies always preferred cuelure over a sugar rich source, unless the maxillary palps were excised.

## Introduction

Tephritid flies are among the most severe agricultural and horticultural pest insects responsible for tremendous economic losses worldwide (Clarke et al., 2011). They have a propensity to expand their distribution range, which is likely to further increase due to climatic changes (Goergen et al., 2011). In tephritids, often referred to as “true fruit flies”, females use their strong ovipositor to puncture the skin of intact and growing fruits to lay their eggs. Whilst oviposition “stings” may cause local mold growth, most of the damage is caused by larval feeding during their development inside the fruit, causing it to rot and become unmarketable. In spite of being endemic to Australia, the highly polyphagous Queensland fruit fly, *Bactrocera tryoni*, is considered one of – if not – the worst horticultural pests in this country attacking a large variety of fruit crops (Drew et al., 1978; Bateman, 1991; Hancock et al., 2000). *B. tryoni* has also been accidentally introduced to the Pacific islands (Bateman and Arretz, 1973) where it poses a global threat to agriculture and horticulture.

In the last 50 years, substantial efforts have been invested into identifying visual and olfactory attractants to implement in “lure and kill” strategies as part of sustainable pest management programs. While *B. tryoni* females are attracted to a three-component mixture of fruit derived esters (Cunningham et al., 2016), no effective lures have been developed so far. Male flies, on the other hand, are strongly attracted to the phytochemical compound raspberry ketone and its synthetic analog called “cuelure”. When consumed by males, cuelure is metabolized and accumulated in the rectal gland as raspberry ketone, which is subsequently released together with endogenous pheromones, exacerbating their attractiveness to females compared to males that have not ingested cuelure (Kumaran et al., 2014). Consequently, cuelure used in conjunction with insecticides has proven to be a useful attractant for monitoring and controlling purposes (Monro and Richardson, 1969; Bateman and Arretz, 1973; Dominiak et al., 2003).

Considering the monumental impact of tephritids as pests, and given their well-known odor-driven behavior, there are surprisingly few studies on the olfactory system of these flies. In a recent electroantennogram (EAG) study, it was shown that cuelure elicits no response in male or female *B. tryoni* antennae (Siderhurst et al., 2017). However, the authors found that raspberry ketone trifluoroacetate, a fluorinated analog of cuelure, evoked strong antennal responses in both sexes. Activation of antennal receptors by the hydrolysis product of trifluoroacetate rather than the actual test stimulus was given as a possible explanation for these responses (Siderhurst et al., 2017).

In this study, we investigated whether attraction to cuelure may be mediated by olfactory receptors located on other organs than the antennae, namely the maxillary palps. Maxillary palps are mouth parts which have been shown to accommodate specific receptors responding to behaviorally important odorants in *Drosophila melanogaster* (Dweck et al., 2016). We thus hypothesized that *B. tryoni* may also have receptors located on the maxillary palps selectively responding to cuelure, and that the responses may be sexually dimorphic.

First, we determined whether male *B. tryoni* remain attracted to cuelure in the absence of activation of antennal olfactory receptors. For this purpose, behavioral preferences of male and female *B. tryoni* for cuelure or apple juice, used as an alternative food source, were tested in a two-choice trap assay. Next, the importance of both organs in eliciting a cuelure response was determined in similar experiments using insects for which either the antennae or the maxillary palps were excised. In addition, we complemented the behavioral experiments with electrophysiological and optophysiological studies to confirm the role played by the maxillary palps in cuelure detection, and examined sexual dimorphic responses. Our findings suggest that male flies, but not female flies, primarily use their maxillary palps to detect and respond to cuelure.

## Materials and Methods

### Study Species

We obtained *B. tryoni* Froggatt (Diptera: Tephritidae) as pupae from a continuous culture reared on a carrot-based medium and maintained at the Queensland Department of Agriculture and Fisheries (Brisbane, QLD, Australia) and Agriculture Victoria (AgriBio, Melbourne, VIC, Australia). Adult flies were sorted by sex and housed in screen cages (40 cm × 40 cm × 40 cm) under controlled climate conditions (28°C, 75% R.H., 12:12 h light:dark). Water, sugar, and yeast hydrolysate were provided *ad libitum* until the insects were used in experiments.

### Two-Choice Trap Assay

**FIGURE 1:**
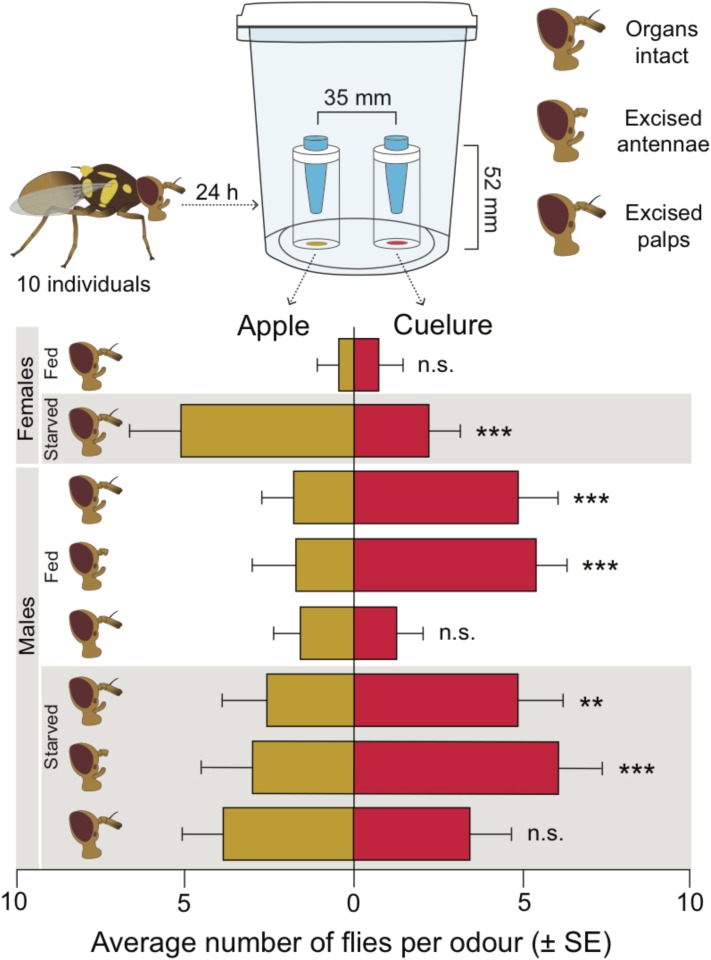
Number of trapped female and male *Bactrocera tryoni* in the two-choice trap assay containing apple juice (yellow) and cuelure (red). A graphic representation of the trap assay and the different options of excised sensory organs is given at the top of the figure. The number of flies trapped in apple juice or cuelure is illustrated with their mean and standard error. n.s., not significant; ^∗∗^*P* < 0.01 and ^∗∗∗^*P* < 0.001. Each treatment consists of 15 trials with 10 flies in each.

We tested the preference of 7–10 days old *B. tryoni* for cuelure (CAS 3572-06-3, Abcam, United Kingdom) vs. apple (pasteurized concentrate, Rynkeby, Sweden) in a two-choice trap assay. The traps consisted of 15 ml glass vials (52 mm × 24 mm) closed with a snap-cap pierced with a 1000 μl pipette tip. The pipette tips were shortened and pushed 30 mm inside the vials to allow for an opening of approximately 4 mm through which the flies could enter (**Figure 1**). A thin layer of paraffin oil (CAS 8102-95-1, Sigma-Aldrich, Sweden) was applied on the end of the pipette tip to ensure that no flies could exit the trap after entering it. The glass traps were secured, 35 mm from one another with odorless dental wax (Heraeus Kulzer, United States), inside an aerated 1 l polypropylene jar. One of the glass vials was loaded with 500 μl of apple juice, and the other vial with 10 μl of 1% (v/v) cuelure diluted in paraffin oil applied on a 15 mm filter paper disc (Grade 1, Whatman, Sweden). A third vial containing a humidified piece of cotton was placed in the jar to supply flies with sufficient water throughout the experiment. Groups of 10 individuals were introduced into the jar and the assay was run for 24 h under controlled conditions (28°C, 75% R.H.), after which the number of flies caught per individual trap was counted. To determine the influence of sex and starvation on *B. tryoni* preference for apple juice or cuelure, we first tested (1) starved females, (2) fed females, (3) starved males, and (4) fed males. Next, we tested (5) fed males with excised antenna, (6) fed males with excised maxillary palps, (7) starved males with excised antennae, and (8) starved males with excised maxillary palps, to determine the role played by both sensory organs in sensing cuelure (*n* = 15 per treatment). Excision of the sensory organs was performed on CO_2_-anesthetized flies by either cutting off the third antennal segment or the entire maxillary palps. In *B. tryoni*, the first two segments, the scape and the pedicel, do not house any chemosensory sensilla (Hull and Cribb, 1997). All flies were allowed to recover for at least an hour after excisions before being used in the trap assays.

### Electrophysiology

Detection of cuelure was tested by performing electrophysiological recordings on both antennae (EAG) and maxillary palps (electropalpogram, EPG) on 7–14 days old *B. tryoni*. Flies were anesthetized using ice and immobilized in 1000 μl pipette tips with only half of the head, including the antennae and palps, being exposed to ambient air. A sharp glass electrode (0.86 mm ID; 1.5 mm OD, Harvard Apparatus, United Kingdom) filled with an electrolyte (0.1 N KCl, 1% PVP) was inserted in the back of the fly’s head and positioned either on the tip of an antenna or maxillary palp. Recordings from antennae (*n* = 8) and palps (*n* = 7) were subsequently obtained from the same insect by repositioning the recording electrode from the antennae to the palps. The signals were acquired using an IDAC-4 (Ockenfels Syntech, Germany) piloted by GC-EAD 2014 software (Version 1.2.5, Ockenfels Syntech, Germany). Stimuli were prepared in odor cartridges consisting of 10 μl of 1% (v/v) dilutions in paraffin oil (Sigma-Aldrich, Australia), applied on 15 mm × 5 mm pieces of filter paper inside glass Pasteur pipettes sealed with 1000 μl pipette tips. Cartridges were “puffed” on the mounted insects using a CS-55 air controller unit (Ockenfels Syntech, Germany) as 0.5 s (0.5 l/min) air pulses applied in a 1.5 l/min continuous humidified and charcoal-filtered airstream. Odor cartridges were tested in a set sequence: (1) paraffin oil (solvent blank), (2) ethyl hexanoate (positive control), and (3) cuelure; at 30 s time intervals and were used for a maximum of four stimulations.

EAG and EPG responses were normalized based on responses elicited by ethyl hexanoate, which was chosen as control due to its low depletion rate in odor cartridges (Andersson et al., 2012) and the consistent responses it elicits in both, antennae (-1.6 ± 0.2 mV) and maxillary palps (-0.5 ± 0.1 mV). Paraffin oil alone elicited only weak EAG responses (-0.26 ± 0.01 mV) and no visible EPG responses.

### Tracing Experiment

Flies were anesthetized on ice and placed inside cut pipette tips allowing the head to protrude at the narrow end. One of the maxillary palps or antennae was excised and neurobiotin crystals (Vector Laboratories, United States) were immediately applied to the fresh wound. The preparation was left in a humidified chamber overnight at 7°C. The following day, the brain was dissected out in 0.01 M phosphate-buffered saline (PBS) and fixed overnight in 4% paraformaldehyde diluted in PBS at 7°C. After careful rinsing in PBS, the brains were incubated for 72 h in mouse monoclonal anti-synapsin (anti-SYNORF1, 1:20; Developmental Studies Hybridoma Bank, Iowa City, IA, United States) and Alexa Fluor 488 conjugated streptavidin (1:500, Life Technologies, Netherlands). The brains were then rinsed again and incubated for 48 h with Alexa 546-tagged secondary antibody (1:500; Invitrogen), to detect anti-synapsin staining. The brains were subsequently rinsed and dehydrated in increasing concentrations of ethanol and cleared in methyl salicylate. Finally, the brains were mounted in methyl salicylate with spacers under cover glasses and scanned with 10× or 20× air objectives using a Zeiss LSM 780 META confocal laser scanning microscope (Zeiss, Jena, Germany).

### Functional Calcium Imaging

We further tested the involvement of the antennae and maxillary palps in sensing cuelure by performing Ca^2+^ imaging on the antennal lobes (following the protocols of Carlsson et al., 2002, 2011). Prior to the recordings, we anesthetized 7–10 days old *B. tryoni* males and females on ice and placed them inside a 1000 μl pipette with the tip cut open to allow the head to protrude at the narrow end. We fixed the head in position with dental wax and removed the mouthparts, including the labellum, pharynx, and maxillary palps. Through this opening we exposed the antennal lobes by removing all the tissue covering the brain. Subsequently, we placed a microscopic cover glass (Ø 13 mm, VWR, Sweden) over the opening in the head to prevent the antenna from getting wet during the rest of the preparation. Next, we bath-applied a drop of a membrane-permeant fluorescent calcium dye (Calcium Green-1 AM, Molecular Probes, Eugene, OR, United States) dissolved in physiological saline with 20% Pluronic F-127 (30 μm, Molecular Probes, Eugene, OR, United States) to the exposed brains and incubated the preparation for about 60 min at 7°C. Finally, the brain was rinsed several times with physiological saline to remove any excessive dye prior to recording.

The imaging set-up consisted of an air-cooled 12-bit slow-scan CCD camera (Olympus U-CMAD3) mounted to an upright microscope (Olympus BX51WI) equipped with a 10× air objective (NA 0,30; Olympus). We excited Calcium Green-1 AM at 475 nm (500 nm SP; xenon arc lamp, Polychrome V, Till Photonics) and detected its fluorescence at 490/515 nm (DCLP/LP). The set-up was operated through the Tillvision 4.0 software (Till Photonics). We tested the antennal responses to 1% (v/v) cuelure, and to 1% (v/v) octanol (CAS 111-87-5, Sigma-Aldrich, Sweden) as a positive control. The odors were diluted in paraffin oil (Sigma-Aldrich, Sweden) and 10 μl of the dilution was loaded onto a rectangular piece of filter paper (15 mm × 5 mm) which was placed into a glass Pasteur pipette. In addition, we tested the antennal response to an odor-cartridge only containing 10 μl paraffin oil on a filter paper as a blank control. While recording the responses in the antennal lobes we ventilated the antennae with a humidified and charcoal-filtered continuous air stream (1 l/min) through a glass tube (5 mm inner diameter) placed approximately 10 mm from the antennae. This glass tube contained one small hole through which an empty Pasteur pipette was inserted and delivered 0.1 l/min air stream. Each recording consisted of a sequence of 50 frames obtained at 4 Hz during which another air stream was blown for 2 s (starting at frame 12) through the odor-cartridge at 0.1 l/min by a computer-triggered puffer device (Ockenfels Syntech, Hilversum, Netherlands) into the continuous stream of air. During stimulation, the air stream was switched from the empty pipette to the odor-laden one in order to minimize the influence of added air volume. We used at least 60 s interstimulus periods to reduce adaptation and tested each odor two to three times in a randomized order depending on the condition of the animal.

### Statistical and Imaging Analysis

For the two-choice trap assay, we first compared the number of females and males, for which the sensory organs were kept intact, trapped in the assay using a generalized linear mixed model (GLMM) with a Poisson error distribution. We included sex, physiological state and an interaction between sex and physiological state as explanatory variables and the replicate of the assay as a random factor. Then, we used GLMMs to compare the preferences among the different sexes and physiological states independently while accounting for the excision of the sensory organs as an additional explanatory variable. Pairwise comparisons across the different excisions of sensory organs were made with a Tukey HSD *post hoc* test with Bonferroni correction. The models were fitted with the lme4 package (Bates et al., 2015), the likelihood ratio tests were performed with the car package (Fox and Weisberg, 2011), and the pairwise comparisons were performed with the multcomp package (Hothorn et al., 2008). The model requirements were checked through estimations of over-dispersion, and inspections of the normality of model residuals and Q–Q plots. When necessary, over-dispersion was corrected for by including a random factor for each observation in the data set. All analyses were carried out in R (v. 3.3.2; R Foundation for Statistical Computing, Vienna, Austria).

Paired *t*-tests were used to compare normalized EAG and EPG responses to cuelure to those of the paraffin oil control. Unpaired *t*-tests were performed to compare male and female responses. To analyze the obtained Ca^2+^ imaging data, we constructed false-color coded images of relative changes of fluorescence intensity during the peak time of activity (frame 16–21). We used the region of highest activity as a proxy for the strongest activated glomerulus and drew circular regions of interest (10 pixels diameter) around the activity foci. An additional region of interest was drawn in an area with minimal activity, which served to correct for bleaching. The mean pixel value within a region of interest was calculated for each time-point in a sequence and exported to Microsoft Excel. In Excel, we first made a temporal median filtering of data over three consecutive frames. Secondly, we calculated the relative fluorescence (d*F*/*F*) where *F* was defined as the mean value of frames 3–10. To correct for bleaching we subtracted the values of the control region of interest from the values of activity region of interest for each recording. A response was finally defined as the mean of frames 16–21 (peak of activity). Responses to cuelure and octanol were compared with responses to the solvent control, containing paraffin oil, using an unpaired *t*-test. Finally, responses to cuelure and octanol were compared.

## Results

### Two-Choice Trap Assay

We found significant effects of sex (GLM: χ^2^ = 10.43, *P* = 0.001), physiological state (GLM: χ^2^ = 16.91, *P* < 0.001), and of the interaction between sex and physiological state (GLM: χ^2^ = 34.45, *P* < 0.001), on the number of females and males trapped in cuelure or apple juice (**Figure 1**). To understand the interaction, we analyzed the sexes individually and found a strong effect of physiological state on the number of trapped females (GLM: χ^2^ = 50.68, *P* < 0.001), which is evidently caused by a lack of response by the fed females and a strong preference for apple juice by starved females (**Figure 1**). For the males, we found significant effects of physiological state (GLM: χ^2^ = 16.36, *P* < 0.001), excised sensory organs (GLM: χ^2^ = 16.12, *P* < 0.001) and of the interaction between physiological state and the excised organs (GLM: χ^2^ = 14.60, *P* = 0.001). This interaction occurred because the excision of the different sensory organs had a strong effect on the number of fed males trapped in cuelure (GLM: χ^2^ = 27.74, *P* < 0.001), but not on the number of starved males (GLM: χ^2^ = 2.98, *P* = 0.225; **Figure 1**). The pairwise comparisons showed that the preference for cuelure by fed males, with and without antennae, did not differ from each other (*P* = 0.987), but significantly differed from the response of males with excided palps (both comparisons *P* < 0.001). For the starved males, none of the pairwise comparisons showed significant differences between the number of trapped flies with or without their sensory organs (all comparisons *P* > 0.345).

### Electrophysiological Evidence of the Detection of Cuelure in Different Sensory Organs

**FIGURE 2:**
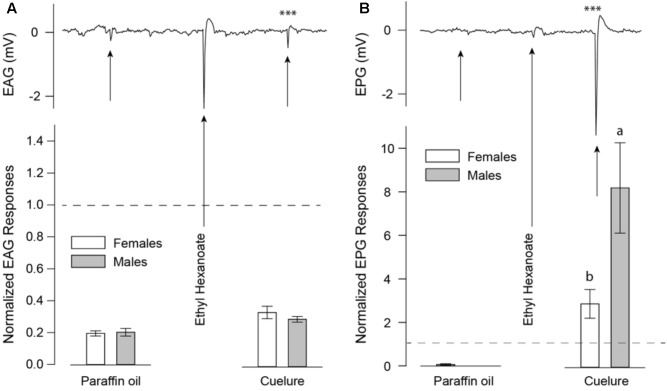
Electroantennogram (EAG) **(A)** and electropalpogram (EPG) **(B)** of *Bactrocera tryoni* responding to paraffin oil as a blank control, ethyl hexanoate as a positive control, and cuelure. Black arrows on the EAG (*n* = 8) and EPG (*n* = 7) traces indicate chemical stimulations. White bars represent normalized responses of adult females and gray bars that of males with their standard errors. Letters above bars indicate statistical differences between male and female responses. Asterisks over the traces depict statistical differences of normalized responses compared with responses elicited by paraffin oil alone (^∗∗∗^*P* < 0.001). Dashed lines indicate baseline response of ethyl hexanoate used as positive control and reference for data normalization.

Detection of cuelure by both antennae (EAG; **Figure 2A**) and maxillary palps (EPG; **Figure 2B**) was verified by electrophysiological recordings. Antennal responses to cuelure differed significantly from those of the solvent control of paraffin oil in male [-0.42 ± 0.07 mV, *P*(cuelure vs. solvent) = 0.001] and female flies [-0.44 ± 0.06 mV, *P*(cuelure vs. solvent) = 0.007]. However, antennal response strength did not differ significantly between sexes (unpaired *t*-test, *P* = 0.34). Stronger responses to cuelure were recorded from maxillary palps in both males [*P*(cuelure vs. solvent) = 0.007] and females [*P*(cuelure vs. solvent) = 0.003], the amplitude of which was, this time, significantly greater in males (-2.93 ± 0.32 mV) than in females (-1.24 ± 0.17 mV, unpaired *t*-test, *P* = 0.02).

### Tracing Experiment

**FIGURE 3:**
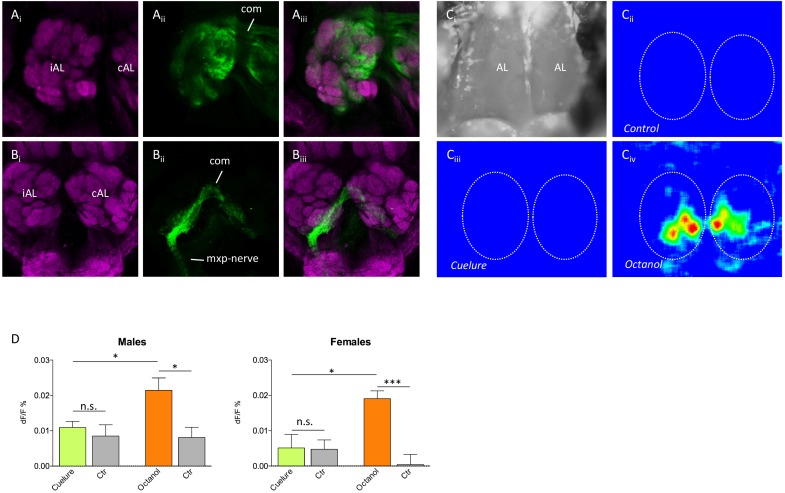
Antennal lobe innervation and odor-evoked activity. Tracing of **(A_i_–A_iii_)** antennal and **(B_i_–B_iii_)** maxillary palp innervation in the antennal lobe (magenta, anti-synapsin staining; green, neurobiotin tracing). The iAL (ipsilateral) is the antennal lobe on the same side as the traced nerve, whereas the cAL (contralateral) only receives innervation via the commissure (com). Maxillary palp nerves (mxp-nerve) only innervate four to five glomeruli in the ventromedial part of both lobes, whereas antennal nerves innervate the remaining about 50 glomeruli. **(C_i_–C_iv_)** False color coded Ca^2+^ imaging responses to control, cuelure, and octanol, respectively. Panel **(C_i_)** shows a grayscale image of the area of the brain recorded from, while panels **(C_ii_–C_iv_)** show the relative change in activity (d*F*/*F*) during stimulation in a single male individual. In panels **(C_ii_–C_iv_)**, the outlines of the antennal lobes are depicted by the dotted lines. The responses in panels **(C_ii_–C_iv_)** have identical intensity scales. **(D)** Mean ± SEM of the relative change in activity during odor stimulation. For cuelure and octanol, the response in the strongest activated glomerulus was used and compared with a solvent control response (paraffin oil). n.s., not significant, ^∗^*P* < 0.05, ^∗∗∗^*P* < 0.001 (unpaired *t*-test).

Neurobiotin back fillings from the antennae and maxillary palps showed that about 50 glomeruli were innervated by antennal sensory neurons and four to five glomeruli were innervated by maxillary palp neurons (*n* = 4 of each; **Figures 3A_i_–A_iii_,B_i_–B_iii_**). Furthermore, both the antennal nerve and the maxillary nerve connected the contralateral antennal lobe through a dorsal commissure and innervated the corresponding glomeruli in that lobe.

### Functional Calcium Imaging

To verify that the weak EAG response to cuelure is caused by the lack of responses in antennal olfactory receptors, we used Ca^2+^ imaging and recorded odor-evoked activity in the antennal lobe with the maxillary palps excised (**Figures 3C_i_–C_iv_**). Only very weak responses to cuelure were observed and the responses did not differ from the solvent control in either of the sexes (*P* > 0.05, *n* = 7 males and *n* = 5 females; **Figure 3D**). Responses to the positive control, octanol, differed significantly to both control and cuelure in both sexes (*P* < 0.05, *n* = 7 males and *n* = 5 females).

## Discussion

In this study, we used a combination of behavioral and physiological assays to demonstrate that *B. tryoni* rely more heavily on the maxillary palps than the antennae to detect and respond to cuelure. We found that male flies with intact olfactory organs consistently preferred the smell of cuelure over apple juice regardless of satiety status. By contrast, female flies exhibited no clear preference when fed, and were more attracted to apple juice when starved. Cuelure attraction of fed and starved males was maintained even when their antennae were excised but was lost as a consequence of maxillary palp removal (**Figure 1**). Consequently, our behavioral experiments suggest that maxillary palps accommodate the essential sensory accessories conferring sensitivity to cuelure in this fly species. Unlike Siderhurst et al. (2017), our EAG recordings revealed that antennae from both males and females responded – though weakly – to cuelure (**Figure 2A**). In line with our behavioral assays, much stronger responses to cuelure were obtained from the maxillary palps (**Figure 2B**). More importantly, maxillary palp responses to cuelure were found to be significantly stronger in males than in females consistent with the greater attraction to cuelure exhibited by the former.

To verify that cuelure is primarily activating receptors on the palps rather than on the antennae, we first used Ca^2+^ imaging and recorded odor-evoked activity in the antennal lobe with the maxillary palps excised. We found that cuelure did not evoke a response that differed from the solvent control among olfactory glomeruli in the antennal lobe of both sexes (**Figure 3**). The existing discrepancies between our imaging and electrographic responses, i.e., no response in the antennal lobe but weak response in the antennae, could be due to modifications of the activity in olfactory sensory neurons terminals by, for example, feedback from local interneurons or systemic peptides (Ignell et al., 2009; Root et al., 2011). It is possible that the local interneurons enhanced contrast between stimuli already at the sensory input level and filter out responses to weakly responding odorants. In our tracing experiment, we could show that four to five ventromedial glomeruli in both sexes were innervated by sensory neurons from the maxillary palps, whereas the remaining (∼50) glomeruli receive inputs from the antennae only (**Figure 3**). This implies that four to five different types of olfactory receptor neurons, and possibly receptor types, may be located on the maxillary palps. While our results concords with previous findings in *D. melanogaster* (Dweck et al., 2016), future experiments are needed to demonstrate greater expression of cuelure-specific receptors in males than in female flies.

In addition, we showed that innervation from both the antennae and the maxillary palps is bilateral, i.e., neurons from one maxillary palp or antenna innervate both the ipsilateral and the corresponding contralateral glomeruli, which has also been found in *D. melanogaster* (Vosshall et al., 2000), but other than dipteran species, unilateral innervation seems to be the rule (Galizia and Sachse, 2010). In an attempt to conclude that palp-innervated glomeruli were activated in a sex-specific manner we tried to perform Ca^2+^ imaging exclusively from palp glomeruli by excising antennae and leave the maxillary palps intact. Despite hundreds of trials, no reliable recordings could be obtained, mainly because the attachment of the labellum to the esophagus had to be left intact, thus causing severe movement problems in the antennal lobes during our recordings. Nevertheless, the lack of response from antenna-innervated glomeruli to cuelure indirectly corroborates electrophysiological evidence of palp-mediated detection of cuelure.

Earlier studies in *D. melanogaster* proposed that maxillary palps were primarily involved in taste enhancement due to their low sensitivity (de Bruyne et al., 1999; Hallem and Carlson, 2006; Shiraiwa, 2008). However, Dweck et al. (2016) recently showed that specific ligands including methyl eugenol; another known male-specific attractant in tephritids; could activate the palps with similar sensitivity as in the antenna.

Considering that other studies have shown that *B. tryoni* males are attracted to cuelure over long ranges (e.g., Monro and Richardson, 1969), our study is the first to demonstrate long range attraction to an odorant via maxillary palps in a tephritid fly. An interesting finding was that males preferred cuelure irrespective of satiety status. Feeding of raspberry ketone together with yeast to immature male *B. tryoni* has been shown to increase mating propensity (Akter et al., 2017). However, raspberry ketone is no substitute to sugar and therefore, flies cannot survive by feeding exclusively on cuelure. Thus, we wonder if the strong innate response to cuelure reflects the evolutionary importance of finding raspberry ketone (its natural analog) in nature, over finding perhaps more available feeding resources. As females are much more responsive to cuelure-fed males, it is possible that a strong selective pressure has been put on males to ingest it. For example, in crayfish, it was shown that males prefer a pheromone over food odor even after seven days of starvation (Pecor and Hazlett, 2008). Whilst the ecological meaning of *B. tryoni*’s attraction to raspberry ketone remains poorly understood, our study certainly suggests that screening maxillary palp sensitivity for a wide range of ecologically relevant odorants may lead to the discovery of new candidate attractants or the reconsideration of previously overlooked ones.

The economic importance of *B. tryoni* urges the need for alternative methods to control fruit fly outbreaks. More broadly, the present study highlights the benefits of gaining a deeper understanding of insects’ olfactory system, which may prove invaluable for the development of new pest control strategies.

## Author Contributions

TV and MAC wrote the manuscript. TV, KF, JPC, and MAC designed the experiments. TV performed the behavioral experiments. KF and JPC performed EAG and EPG recordings. MAC performed the tracing experiments. MAC and TV performed the imaging experiments. All authors reviewed the manuscript.

## Conflict of Interest Statement

The authors declare that the research was conducted in the absence of any commercial or financial relationships that could be construed as a potential conflict of interest.
